# Differential Frond Growth in the Isomorphic Haploid–diploid Red Seaweed *Agarophyton chilense* by Long‐term In Situ Monitoring

**DOI:** 10.1111/jpy.13110

**Published:** 2021-02-09

**Authors:** Vasco M.N.C.S. Vieira, Aschwin H. Engelen, Oscar R. Huanel, Marie‐Laure Guillemin

**Affiliations:** ^1^ MARETEC Instituto Superior Técnico Universidade Técnica de Lisboa Av. Rovisco Pais 1049‐001 Lisboa Portugal; ^2^ CCMAR Center of Marine Science University of Algarve Campus Gambelas 8005‐139 Faro Portugal; ^3^ Departamento de Ecología Facultad de Ciencias Biológicas Pontificia Universidad Católica de Chile Santiago Chile; ^4^ CNRS UMI 3614 Evolutionary Biology and Ecology of Algae Sorbonne Universités UPMC University Paris VI Station Biologique de Roscoff CS 90074 Place G. Tessier 296888 Roscoff France; ^5^ Instituto de Ciencias Ambientales y Evolutivas Facultad de Ciencias Universidad Austral de Chile Casilla 567 Valdivia Chile

**Keywords:** evolution, evolutionary stability, isomorphic biphasic life cycle, population dynamics, rhodophyte, shrinkage

## Abstract

Conditional differentiation between haploids and diploids has been proposed to drive the evolutionary stability of isomorphic biphasic life cycles. The cost of producing and maintaining genetic information has been posed as a possible driver of this conditional differentiation. Under this hypothesis, haploids benefit over diploids in resource‐limited environments by halving the costs of producing and maintaining DNA. Spared resources can be allocated to enhance survival, growth or fertility. Here we test in the field whether indeed haploids have higher growth rates than diploids. Individuals of the red seaweed *Agarophyton chilense*, were mapped and followed during 2 years with 4‐month census intervals across different stands within the Valdivia River estuary, Chile. As hypothesized, haploids grew larger and faster than diploids, but this was sex‐dependent. Haploid (gametophyte) females grew twice as large and 15% faster than diploids (tetrasporophytes), whereas haploid males only grew as large and as fast as the maximum obtained by diploids in summer. However, haploid males maintained their maximum sizes and growth rates constant year‐round, while diploids were smaller and had lower growth rates during the winter. In conclusion, our results confirm the conditional differentiation in size and growth between haploids and diploids but also identified important differences between males and females. Besides understanding life cycle evolution, the dynamics of *A. chilense* frond growth reported informs algal farmers regarding production optimization and should help in determining best planting and harvesting strategies.

**Abbreviations**
Aut: Autumn season
C1: Corral pool #1
C2: Corral pool #2
F: female hapoloids
M: male haploids
N1: Niebla pool #1
N2: Niebla pool #2
N3: Niebla pool #3
Spr: spring‐summer season
Win: winter season

Many red algae exhibit an isomorphic biphasic life cycle with their diploids (tetrasporophytes) and dioicous haploids (gametophytes) virtually undistinguishable to the naked eye. The mechanisms sustaining the evolutionary stability of these life cycles are still debated among ecologists and evolutionary biologists. Conditional differentiation among phases has been posed as the promoter of this stability (Hughes and Otto 1999, Scott and Rescan 2016, Bellgrove et al. 2020) and implies a niche exploitation differentiation between entities in shared habitat. Applied to biphasic life cycles, conditional differentiation limits the competition among ploidy phases. This could be expressed as differences in seasonality, habitat use (e.g., intertidal vs. subtidal) or, in homogeneous stable environments, to ontological differences between haploids and diploids (e.g., one phase is more successful at the adult stage while the other is more successful at the stage of spore or young germling; see Hughes and Otto 1999). Observations of conditional differentiation in seaweeds have accumulated during the last decades (Hannach and Santelices 1985, Luxoro and Santelices 1989, Destombe et al. 1993, Gonzalez and Meneses 1996, Thornber and Gaines 2004, Garbary et al. 2011, Guillemin et al. 2013, Vieira et al. 2018a,b, Bellgrove and Aoki 2020). Besides direct observations, indirect evidence supporting conditional differentiation has also emerged. Theoretically, such differentiation leads to a spatial and/or temporal partition of niches (Thornber and Gaines 2004, Vieira and Santos 2010, 2012a,b, Vieira and Mateus 2014) matching the uneven field abundances between ploidy phases generally reported in red algae, as in the genera *Chondrus* (Craigie and Pringle 1978, Bellgrove and Aoki 2008, Garbary et al. 2011, Krueger‐Hadfield et al. 2013), *Gelidium* (Carmona and Santos 2006), *Gracilaria* (Engel et al. 2001, Guillemin et al. 2008), *Iridaea* (Luxoro and Santelices 1989), *Mazzaella* (Mudge and Scrosati 2003; Thornber and Gaines 2003, 2004, Scrosati and Mudge 2004, Dyck and DeWreede 2006) and *Sarcothalia* (Otaíza et al. 2001).

Complementing the ecological differentiation between phases hypothesis, the nutrient limitation hypothesis proposes that haploids benefit from spending half the resources in DNA replication and maintenance when resources are limiting (Lewis 1985, Mable 2001). Various abiotic essential resources (e.g., nitrogen, phosphorus, inorganic carbon and light) can limit macroalgal growth. Moreover, position along the shore and stand density have a major impact on individual’s capacity of acquisition of energy. Bathymetric gradients (influencing light intensity and spectral composition, proportion of time underwater) directly affect photosynthetic efficiency, desiccation and growth (Ganzon‐Fortes 1997, Sagert et al. 1997, Silva et al. 1998, Gévaert et al. 2003) and was demonstrated for intertidal red algae at our survey location (Gomez et al. 2004, Varela et al. 2006, Andrés et al. 2006). Since the limitation may also be thermodynamic, it should be more correct to generalize this hypothesis as a “Resource Limitation Hypothesis”. The cost of producing and maintaining genetic information was indeed proven determinant for the evolution of unicellular organisms (Mable 2001, Lynch and Marinov 2015), while other studies suggested this may also be so for some macroalgae (Destombe et al. 1993, Reef et al. 2012, Neill et al. 2018). Accordingly, a field study we conducted on *Agarophyton chilense* (previously named *Gracilaria chilensis*; Gurgel et al. 2018) showed that, under stressful conditions demanding specific and efficient resource allocation, adult haploid females had a higher survival than adult diploids (Vieira et al. 2018a) while the adult haploid males showed increased fecundity compared to any other stage (Vieira et al. 2018b). Furthermore, it was also observed that diploid germlings had a higher survival than haploid germlings (Vieira et al. 2018a), most likely due to maternal care by the haploid females passing resources on to their diploid progeny (Kamiya and Kawai 2002). All together, these observations suggest that a resource limitation hypothesis may justify both a haploid advantage on the adults (directly) and a diploid advantage on the sporelings (indirectly, through haploid maternal care). Consequently, a resource limitation hypothesis may drive the conditional differentiation between ploidy phases required for the evolutionary stability of isomorphic biphasic life cycles.

If haploids do benefit from spending significantly fewer resources on the production and maintenance of DNA, these savings can be directed to enhanced growth rates, resulting in higher growth rates of haploids than diploids. However, previous tests comparing frond growth provided somewhat discordant results. In a short‐term study on *Agarophyton chilense* under controlled laboratory conditions (Guillemin et al. 2013), diploid apical meristems grew faster than haploids. However, under reduced light and salinity, growth of entire individuals (germlings and juveniles) was higher in haploids than in diploids. The growth of *Agarophyton vermiculophyllum* (as *Gracilaria vermiculophylla*) was tested by Abreu et al. (2011). At 30 d old, diploid sporelings already showed initial sizes twice as large as the haploid sporelings; however individual size at the end of the experiment depended not only on ploidy but also on temperature, irradiance and photoperiod. The growth of apical meristems excised from adult fronds was also tested. In this case, no significant differences were found between haploids and diploids. In an experiment with *A. vermiculophyllum,* the level of difference between haploid females and the other two life cycle stages in terms of growth depended on site and nutrient level (Krueger‐Hadfield and Ryan 2020). In two experiments with apical meristems of *Gracilaria dura*, diploids grew more than haploids when the last 2 cm were excised (Sambhwani et al. 2020), but haploids grew more than diploids when the last 5 cm were excised (Gupta et al. 2011). Neill et al. (2018) found that female fronds of *Sarcothalia lanceata* were longer whereas haploid males were wider and increased their widths faster. In *Chondracanthus chamissoi* diploid germlings grew faster than haploid germlings, during long day lengths (Gonzalez and Meneses 1996). However, *Gelidium sesquipedale* diploid germlings grow faster than haploids germlings irrespective of photoperiod (Carmona and Santos 2006). Besides the diversity of environmental conditions tested, several factors intrinsic to the growth process may contribute to a lack of consensus. First, drawing conclusions from comparisons involving germlings and/or juveniles may be difficult since the simultaneous effects of resource limitation and maternal care could offset each other. The results of growth experiments seem contradictory when testing entire individuals compared to testing just fragments containing the apical meristems. Fronds may have allometric scaling and thus, besides length, width should also be tested. Adequate testing requires growth metrics well suited to describe the frond growth dynamic. The use of Relative Growth Rates (i.e., growth measured as % · d^−1^), often chosen to represent frond growth dynamic (Abreu et al. 2011, Gupta et al. 2011, Oliveira et al. 2012, Ursi et al. 2013, Araújo et al. 2014, Mantri et al. 2020, Sambhwani et al. 2020), may be problematic as it assumes infinite growth, disregarding that fronds actually show a size‐dependent growth toward an asymptotic maximum size. Most likely sources of bias are too large census intervals, and different groups starting from different initial sizes and/or tending to different asymptotic sizes.

In this work, we tested for growth differences between *Agarophyton chilense* gametophyte (haploid male and female) and tetrasporophyte (diploid) fronds monitored in situ for two consecutive y. In this case, net change in frond size resulted from the conjugation of approximately continuous slow growth with episodic breakage of large frond fragments. We then discuss how the knowledge brought about by our study may (i) support existing theories about the evolution of isomorphic biphasic life cycles, and (ii) help planning harvesting strategies for this commercially exploited macro‐alga.

## METHODS

### Demographic data

*Agarophyton chilense* is a red seaweed occurring in the shallow subtidal/intertidal area along the Chilean shore. Individuals are fixed to the rocky bottom by a holdfast and have the ability to survive and re‐grow new fronds after older fronds are lost (Guillemin et al. 2013). The species presents a typical isomorphic haplodiplontic life cycle in which both tetrasporophytes (diploids) and dioicous gametophytes (haploids) have a substantial somatic development. Our three‐stage conceptual model considered both ploidy stages (tetrasporophytes and gametophytes) and distinguished male and female gametophyte sexes. Adult gametophytes produce gametes by mitosis and male gametes are released into the water column. Fertilization occurs on the female thallus, forming a fruiting structure called the cystocarp. There, the zygote undergoes successive mitotic divisions and develops as a short‐lived diploid epiphytic stage (Searles 1980, Kamiya and Kawai 2002), called carposporophyte. Our model considered this stage implicit within the gametophyte female as it was impossible to individually follow carposporophytes over time in the field. The carposporophytes release diploid spores (carpospores) into the environment and these were included in the model as female spore output (see Vieira et al. 2018b). Carpospores settle on rocky substratum and develop into tetrasporophytes that produce haploid spores (tetraspores) after meiosis. After release, tetraspores grow into male and female gametophytes, thus closing the cycle.

To determine the demography of this species, all individuals in 5 intertidal rock‐pools (“Corral 1,” “Corral 2,” “Niebla 1,” “Niebla 2” and “Niebla 3”) were monitored. The sites Corral (39°52′27″ S, 73°24′02″ W) and Niebla (39°55′47″ S, 73°23′57″ W) were located along the margins of the Valdivia river estuary (Fig. S1 in the Supporting Information). All 5 pools corresponded to the stands situated higher in the intertidal and uncovered at the same time. The Niebla stands were in rock‐pools preserving some amount of water during low tide (Fig. S1). Their areas were 0.4 m^2^, 0.7 m^2^ and 0.7 m^2^ respectively for Niebla 1, Niebla 2 and Niebla 3. The Corral stands were on rocky platforms presenting a gentle slope and the individuals desiccated on the bare rock during low tide (Fig. S1). Their areas were 0.9 m^2^ and 0.6 m^2^ respectively for Corral 1 and Corral 2. Desiccation and UV exposure were particularly harsh at Corral 2, with fronds experiencing high mortalities (Vieira et al. 2018a). Sampling occurred from October 2009 to February 2012 at 4‐month intervals. The interval from October to February encompasses the austral spring and summer, a period during which the intertidal stands are subject to intense temperatures and UV, leading to high mortality and low investment in sexual reproduction (Vieira et al. 2018a,b). The interval from February to June mostly encompasses the austral autumn characterized by milder environmental conditions, higher survival (Vieira et al. 2018a) and higher fecundity (Vieira et al. 2018b). The interval from June to October mostly encompasses the austral winter, a period characterized by the lowest sunlight and temperatures. Mortality increased strongly for the smaller while decreasing for the larger fronds (Vieira et al. 2018a).

All individuals within each rock‐pool were mapped and had a small fragment of their thallus collected. Males (M, *n* = 127), females (F, *n* = 305) and tetrasporophytes (D for diploids, *n* = 238) were identified using observed reproductive structures under a binocular microscope (see fig. 1 in Vieira et al. 2018b), and the sex‐specific molecular markers for the remaining vegetative individuals (Guillemin et al. 2012). Each individual was followed from birth to death, with its frond length and diameter recorded at each census. The volume (*v*) of a cylinder of similar length and diameter was used to estimate frond biomass (as dry weight) from an empirical relationship (*r* ^2^ = 0.877; *n* = 281; *P* < 0.0001). Given the bushiness of the thalli, this is the best non‐destructive method to estimate thallus biomass (Stagnol et al. 2016). Every individual absent after four months was re‐checked in the next census for confirmation and considered as dead when missing. Overall, 6,062 size observations were obtained. Following individuals from birth to death allowed keeping track of their age. The maturity state of each frond at each time was also recorded.

### Growth and breakage rates

**Fig. 1:**
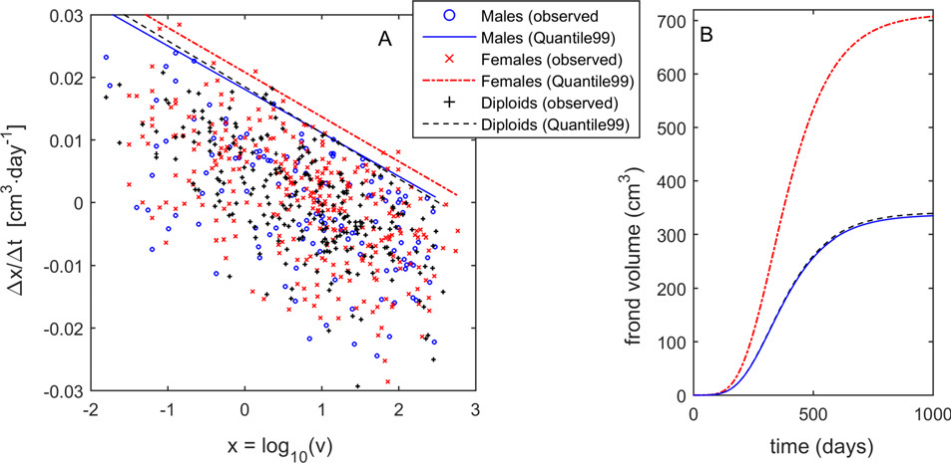
Frond growth of *Agarophyton chilense* estimated for the instantaneous growth model and compared among life cycle stages. (A) Maximum instantaneous growth rates (∆*x*/∆*t*) estimated for male gametophytes (*n* = 127), female gametophytes (*n* = 305) and tetrasporophytes (*n* = 238), using Quantile Regressions for the 99% quantile. (B) projections of frond potential size (i.e., growing without breaking) performed by running the instantaneous growth model for each stage. [Colour figure can be viewed at wileyonlinelibrary.com]

Fronds experienced non‐linear growth, with the growth rate *R* = *v_t_*_+∆_ *_t_*/*v_t_*. The use of instantaneous growth rates, achieved with the transformation *x* = log_10_(*v*), linearized the model, allowing the observed *R* = 10^∆^ *^x^* to be fit to the modelled *R* = 10^(^ *^a^* ^+^ *^bx^* ^)∆^ *^t^*. Reworking, the fitting equation became ∆*x*/∆*t* = *a* + *bx* (Fig. 1A). The intercept “*a*” represented a standard for comparing growth rates without size limitation, that is, during the initial exponential growth phase, when *x* ≈ 0, *v* = 1 cm^3^ and *R* = 10*^a^* ^∆^ *^t^*. The slope “*b*” was always negative, representing the growth rate size‐dependent limitation, that is, the smoothing of the initial exponential growth until the maximum size is attained.

Fronds are also subject to breakage. The decrease in frond size due to the loss of fragments resulted in ∆*x* < 0 and consequently *R* < 1. Under field conditions, short census intervals allow clear separation of observed growth (i.e., fronds with small positive ∆*x*) from breakage (i.e., fronds with larger negative ∆*x*). The 4‐month intervals used in this study (∆*t* = 122 d) complicated such distinction, with the net change in frond size resulting from a combination of both processes. Even when ∆*x* > 0, these fronds may still have broken. Hence, accessing growth and breakage demanded an elaborate data analysis. We considered the fronds showing the largest net size increments as ones that did not break in between census. These corresponded to the observations closest to the upper boundary to the ∆*x*/∆*t* plot against *x* (Fig. 1). This upper boundary was estimated by Quantile Regression (Zhang et al. 2005, Creed et al. 2019), having been used the 99% quantiles.

The ANCOVA procedure was used to compare Quantile regressions within the factors stage (three levels: male gametophytes (M), female gametophytes (F) and tetrasporophytes (D)), season (three levels: Aut, Win, Spr) and site (five levels: C1, C2, N1, N2 and N3). To facilitate the presentation of the results, these factors were tested in a hierarchic (nested) design: (1) differences among stages, (2) differences among seasons within each stage, and (3) differences among sites within each stage × season combination. This final test provided the *a* and *b* coefficients of the instantaneous growth model to be used in the future Individual Based Model (IBM).

Following the ANCOVA protocol, the tests started by accessing the differences among the slopes (*b*) estimated for each level. Their significances were inferred by permutation tests (original data plus 9,999 randomizations), since the former use of overlapping confidence intervals was proved biased (Vieira and Creed 2013a,b). Levels exhibiting non‐significant difference for slopes were attributed a common pooled slope. Then, the tests proceeded to the estimation of the intercepts (*a*) and the significances of their differences. These significances were also estimated using permutation tests. Slope (*b*) estimates for the factor stages were preserved and passed down the hierarchic ladder to the remaining factors, due to limited accuracy, for use in the estimation of the intercepts.

For each randomization simulating the null hypothesis (H_0_: no differences among levels), observations were randomly reassigned among groups to eliminate group differences. Then, a new intercept was estimated for each group by imposing the pooled slope to a re‐estimation of the Quantile Regression. Significances were estimated as one‐tailed because we are only interested in the random luck of falling above the 99% quantile, and not below the 1% quantile on the opposite extreme of the sampled bivariate distribution.

The observations below the maximum growth boundary lines (as in Fig. 1) corresponded to fronds that grew slower and/or broke. We estimated the *Β* (Beta) probability distribution of the difference *d* between potential (i.e., maximum possible = *a* + *bx*) and realized (i.e., observed = ∆*x*/∆*t*) growth. Because this distribution is constrained within {0,1}, its estimation required replacing *d* by *d* + 0.01 and then doing the reverse path back to d. Fronds showing large *d* (=*a* + *bx *− ∆*x*/∆*t*) were considered broken along the 4‐month census interval. We further hypothesized that this probability of a frond to break was, above all, dependent on its size. Hence, in a second test, distinct *Β* (Beta) probability distribution of *d* were estimated for distinct levels of potential frond size, that is, the predictable size of a frond had it grown without ever breaking: *x_p_* = *x* + (*a* + *bx*)∆*t*.

### Model validation

The frond growth differences among stages may also be inferred by comparing differences in their slopes/intercepts as other quantiles are considered (see Cade and Noon 2003). Hence, besides the 99% quantile, we also tested for differences among stages using the 25%, 50% and 75% quantiles. For validation of the Individual Based Model (IBM) of *A. chilense* growth the forecasted final frond increment was compared to the observed frond increment.

## RESULTS

### Growth rates

The finite growth rate of fronds was *R* = 10^(^ *^a^* ^+^ *^b^* ^∙^ *^x^* ^)∆^ *^t^*, were *a* is the size‐standardized instantaneous growth rate, *b* the size‐dependent change in growth, and *x* = log(*v*) is frond size. First, the overall effect of life cycle stage on frond growth rates was tested using the ANCOVA design. The Quantile regressions applied to each stage yielded significant (*P* < 0.001), but similar slopes of *b_M_* = −0.007, *b_F_* = −0.0071 and *b_D_* = −0.0074, for the male gametophytes, female gametophytes and tetrasporophytes, respectively (Fig. 1A; *P_M_*_≠_ *_F_* = 0.599, *P_M_*_≠_ *_D_* = 0.878 and *P_F_*_≠_ *_D_* = 0.5902). Thus, a pooled slope of *b* = −0.0073 was used to determine the significance of differences between intercepts for the remaining analysis. The intercepts estimated for life cycle stages were *a_M_* = 0.0185, *a_F_* = 0.0212, and *a_D_* = 0.0185 (Fig. 1A), with the significantly largest intercept for female gametophytes (*P_M_*_≠_ *_F_* = 0.079, *P_M_*_≠_ *_D_* = 0.865 and *P_F_*_≠_ *_D_* = 0.004). To illustrate the consequences of such differences, time‐series of frond growth were projected considering life cycle stage (Fig. 1B). These forecasts suggest that *A. chilense* fronds took about 2 years to reach their maximum sizes, with the female gametophytes attaining sizes (≈700 cm^3^) about twice as large as the male gametophytes and tetrasporophytes (≈350 cm^3^). Note that these results refer only to 99% of the population, with larger individuals representing the remaining 1%. In fact, two out of the 1,434 male observations where larger than 350 cm^3^ with one even attaining 792 cm^3^. Also, two out of the 2,113 female observations where larger than 700 cm^3^ with one even attaining 2,215 cm^3^. Finally, six out of the 2,515 tetrasporophyte observations where larger than 350 cm^3^ with one even attaining 1,039 cm^3^.

**Fig. 2:**
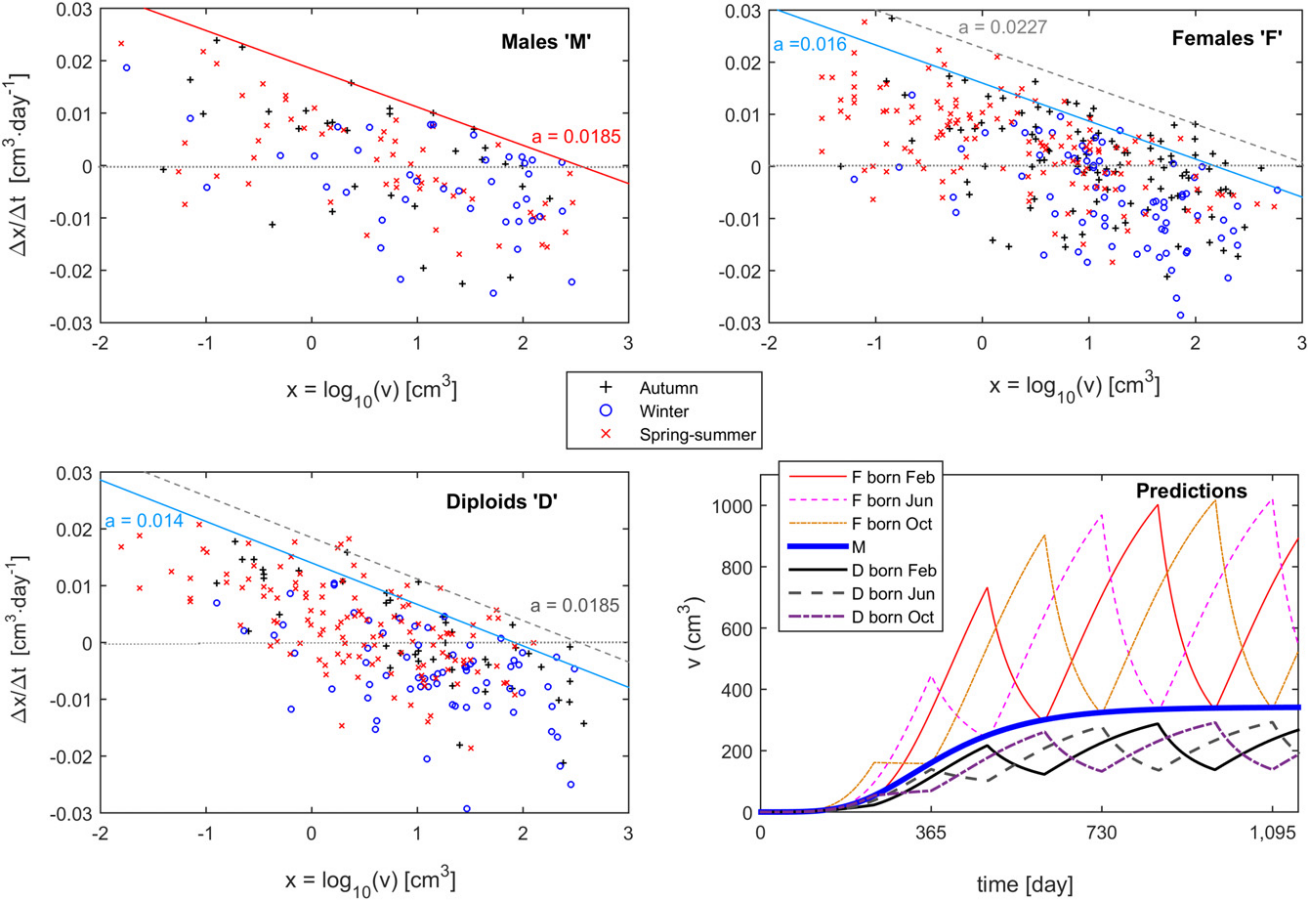
Seasonal dynamics of *Agarophyton chilense* frond growth, as instantaneous growth rates (∆*x*/∆*t*), for three life cycle stages Haploid males, Haploid females and Diploids observed during autumn, winter and spring‐summer, and their fitted boundary lines: haploid females and diploids during the winter (solid lines in Females and Diploids panels), haploid females and diploids during spring‐summer and autumn (dashed gray line), haploid males during all seasons (solid line in Males panel). The horizontal dotted line separates positive from negative ∆*x*/∆*t*. Panel (Predictions) shows the time‐series of potential frond size (i.e., growing without breaking) with germination occurring at the start of the three different seasons. [Colour figure can be viewed at wileyonlinelibrary.com]

Second, we tested for effects of season on growth rate within each life cycle stage. Fronds generally grew more during the warmer, sunnier seasons (spring‐summer) than during the cooler, darker winter. Hence, the observations from the warmer seasons and their fitted lines occurred mostly above those of the cooler season (Fig. 2). However, male fronds seemed to be un‐affected by season (Fig. 2). Over the yearly cycle, the faster diploid growth during the favorable season (corresponding to *a* = 0.0185 in Fig. 2), was only as good as the regular male growth (also *a* = 0.0185 in Fig. 2). Our novel methodology allowed the identification of seasonal shrinkage. The female and diploid fronds, although larger during the favorable spring‐summer and autumn seasons, never obtained sizes beyond this season‐specific asymptotic size, identified by their season‐specific x axis intercept. However, when the winter arrived, these larger fronds had sizes beyond the asymptotic size (=unsustainably large) of this unfavorable season. All observations during winter of fronds initially larger than their winter *x* axis intercept showed size decreases, demonstrating that, during winter and beyond that asymptotic size, growth was impossible and deterioration inevitable. Some of those fronds were on top of the boundary line, meaning that they probably did not break but shrunk back toward their winter x axis intercept. Shrinkage corresponds to progressive decrease in size and biomass resulting from bleaching and cell senescence, as opposed to a sudden loss due to breakage. While small and medium‐sized fronds tended to grow, all the larger fronds effectively decreased in size. The males, on the other hand, neither grew beyond the size of their fixed x axis intercept nor shrunk back. To illustrate such seasonality, time‐series of frond growth were projected considering life cycle stage and season, starting at different seasons (Fig. 2). Besides the seasonal oscillations, this forecast showed that females tended to grow to larger sizes, close to 1,000 cm^3^, due to a higher warmer seasons intercept “*a*”. Diploid fronds, however, never grew as fast nor became as large as haploid fronds from the same cohort, whether male or female.

**Fig. 3:**
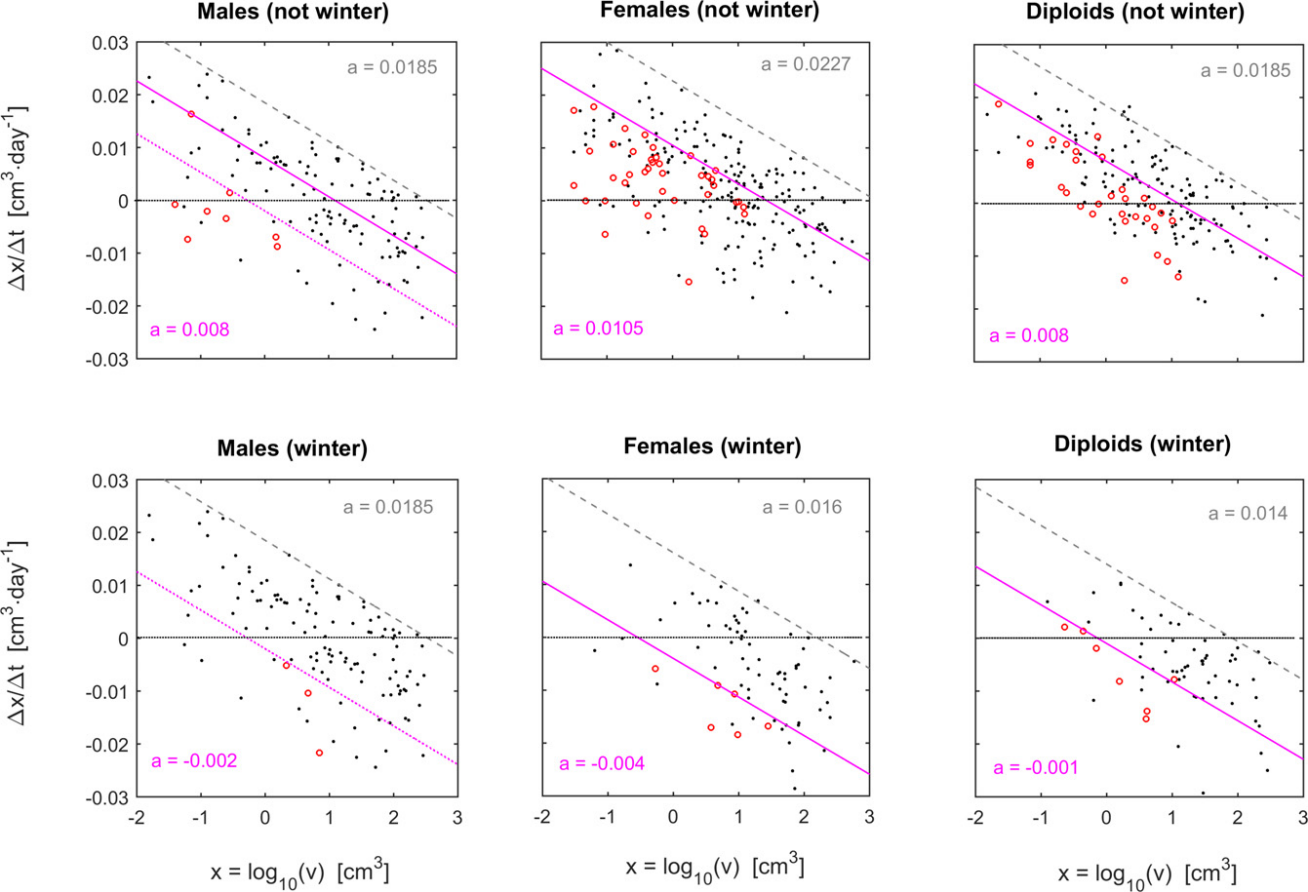
Spatial dynamics of the frond growth of *Agarophyton chilense*. Black dots and dashed gray lines represent sites C1, N1, N2 and N3. Circles and solid lines represent site C2. Comparisons between sampling areas were made for each life cycle stage and seasons. Magenta dotted line represents haploid males in C2 during winter. The horizontal gray dotted line separates positive from negative ∆*x*/∆*t*. [Colour figure can be viewed at wileyonlinelibrary.com]

Third, we tested for site effects on frond growth rates (within each stage × season combination). Site “C2” was particularly unfavorable for size/growth when compared to all other sites (Fig. 3). Fronds in “C2” shrunk even when of moderate size and never attained the large sizes observed elsewhere. Winter was particularly unfavorable for fronds in “C2,” with hardly any growth and virtually unavoidable shrinkage for any reasonably sized frond, even so for haploid males.

**Fig. 4:**
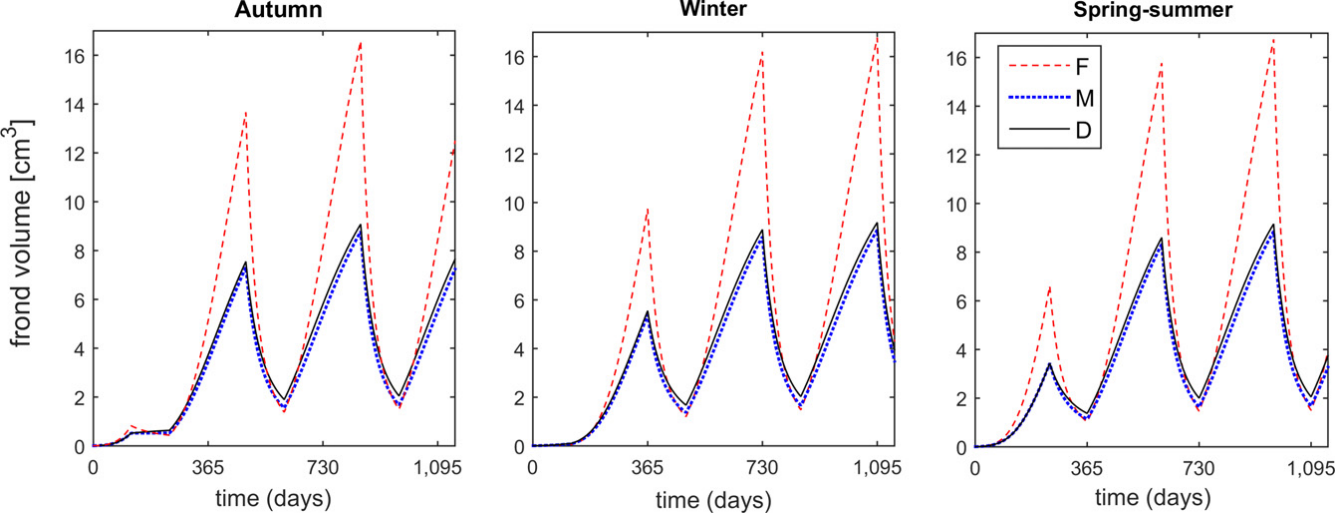
Forecasted potential frond size (i.e., growing without breaking) of *Agarophyton chilense* at site C2 for three life cycle stages (haploid males, M: dotted lines; haploid females, F: dashed lines; and Diploids, D: solid lines), germinated during different seasons: autumn, winter and spring/summer. [Colour figure can be viewed at wileyonlinelibrary.com]

Frond size was forecasted considering life cycle stage, germination season and site. At Niebla and site “C1,” the forecasts correspond to the trajectories shown in Figure 2D. At site “C2,” modeling showed abnormally low growth rates and frond sizes, as well as a differentiation favoring haploid females (Fig. 4). Female fronds in “C2” did not grow beyond 17 cm^3^, but males and diploids did not even grow beyond 8 cm^3^. This very weak frond growth in C2 complements very high mortalities at this same site, as observed by Vieira et al. (2018a).

Complementary factors “age” and “maturity state” (defined as in Vieira et al. 2018a) had no effect on the growth potential of *Agarophyton chilense*. The slopes and intercepts estimated for fertile and unfertile fronds were identical (*P* = 0.69 and *P* = 0.351, respectively). The slopes estimated for germlings (g), juveniles (j) and adults (a) were also identical (*P_g_*_=_ *_j_* = 0.851, *P_g_*_=_ *_a_* = 0.539 and *P_j_*_=_ *_a_* = 0.705); as were their intercepts (*P_g_*_=_ *_j_* = 0.478, *P_g_*_=_ *_a_* = 0.418 and *P_j_*_=_ *_a_* = 0.256).

### Breakage rates

**Fig. 5:**
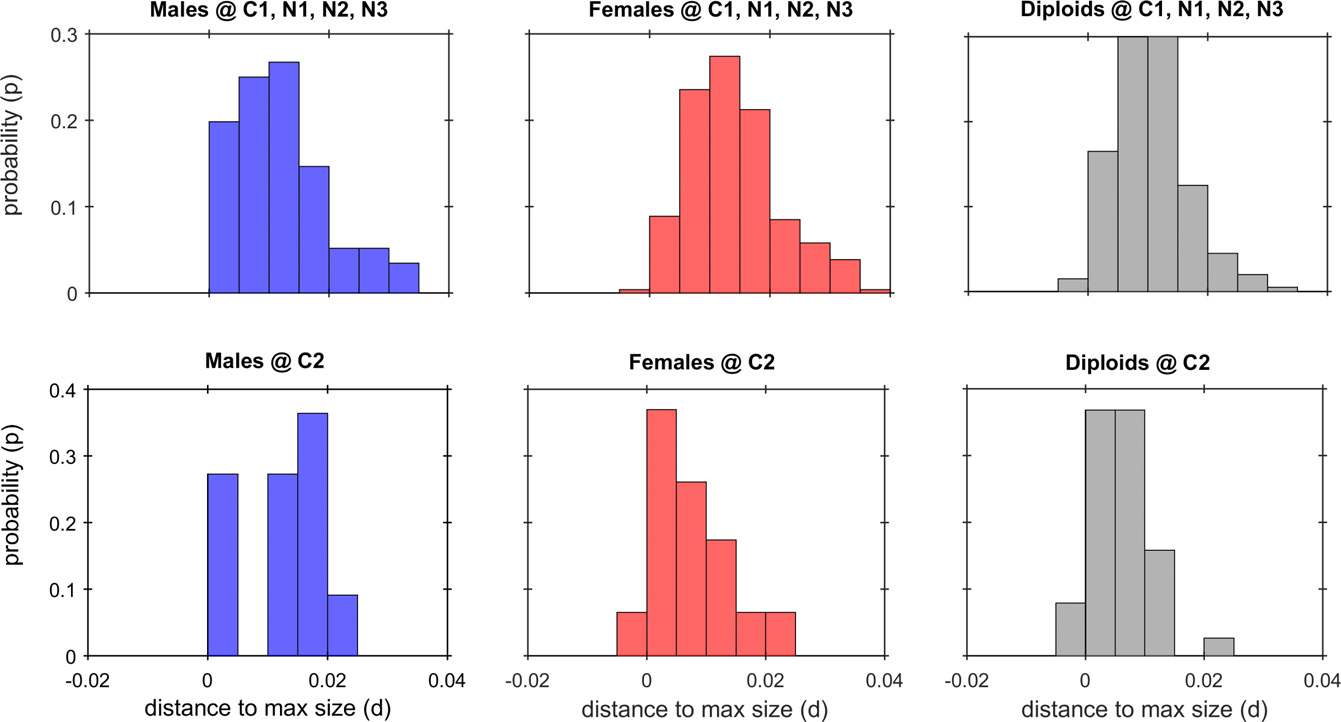
Probability (*P*) distribution of frond size in *Agarophyton chilense* below their size potential ‐ that is, the difference between potential and realized size (*d*) ‐ depending on stage haploid males (M), haploid females (F) and Diploids (D) and site. [Colour figure can be viewed at wileyonlinelibrary.com]

Most fronds never grew to their full potential size (see Figs. 1, 2, 3). The fronds showing a high deviation from full potential size, most likely broke sometime along the 4‐month census interval (Fig. 5). Since larger fronds are more prone to hydrodynamic stress, we formulate the hypothesis that females broke more than males and diploids because they were usually much larger, and not because their tissue was weaker and more prone to breaking. Likewise, fronds in the sites C1, N1, N2 and N3 broke more than the fronds in C2 also because they were usually much larger.

**Fig. 6:**
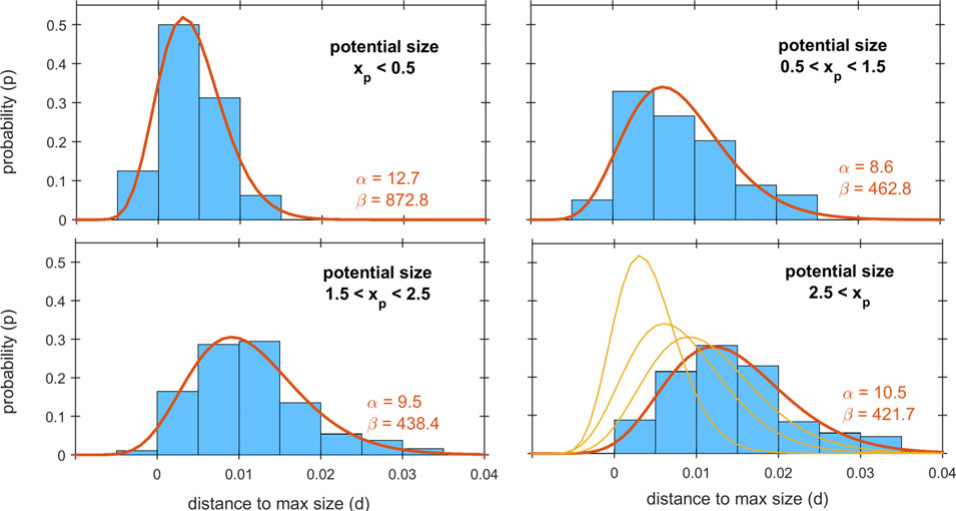
Probability (*P*) distribution of *Agarophyton chilense* fronds sized below their maximum potential ‐that is, the difference between potential and realized size (*d*) ‐ depending on potential size (*x_p_*). The thick lines are the Beta distribution with its α and β parameters. The bottom‐right panel also shows in thinner lines the Beta distributions from previous panels. [Colour figure can be viewed at wileyonlinelibrary.com]

The hypothesis raised above was confirmed by relating d with the potential frond size *x_p_* = *x_t_* + (*a* + *bx_t_*)∆*t* ‐ that is, predictable size if grown at the fastest observed rate and without ever breaking ‐ irrespective of stage or site. As the potential frond size (*x_p_*) increased, so did the difference (*d*) between potential size and realized size (Fig. 6) indicative of breakage. This could not be tested among stages or sites as these factors were not independent from size. Small potential sized fronds (*x_p_* < 1.5, corresponding to *v* < 31.6 cm^3^) consisted only and contained all individuals from site C2. Mid potential sized fronds (1.5 < *x_p_* < 2.5) occurred at all sites except C2. Large fronds (*x_p_* > 2.5, corresponding to *v* > 316 cm^3^) were almost exclusively composed of females. For mid‐sized fronds, the only size class with sufficient replicates for robust testing, breakage (*d*) was independent of life cycle stage (*P*_Chi‐square_ = 0.381), season (*P*_Chi‐square_ = 0.153), age (*P*_pChi‐square_ = 0.875) or maturity state (*P*_Chi‐square_ = 0.583). The relationship between breakage and frond size showed that larger fronds have a higher probability of breaking larger portions of their fronds (Fig. 6). So, irrespective of stage and of initial size, fronds may tend to break to an average final size.

### Model validation

**Fig. 7:**
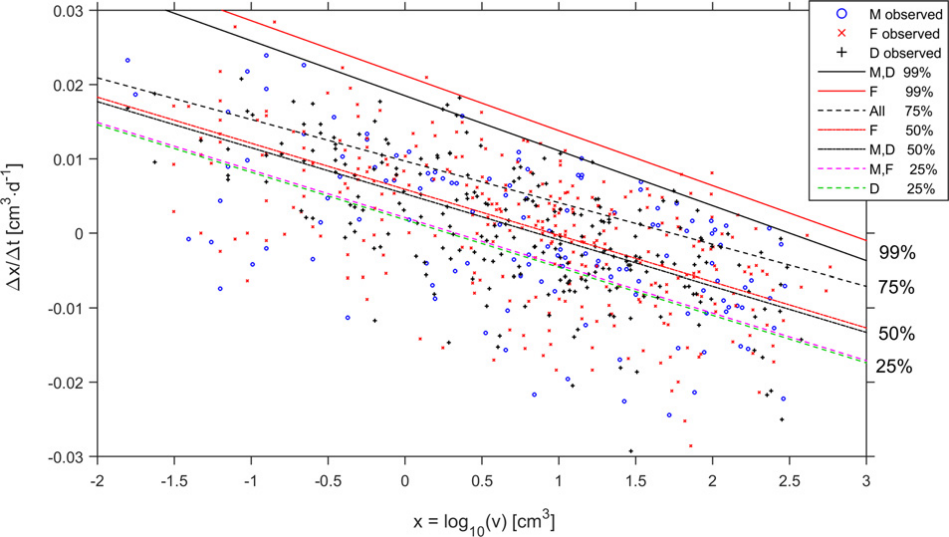
Comparisons among stages and among quantiles for frond growth in *Agarophyton chilense*. Stages were (M) males, (F) females and (D) diploids. Quantiles were 99%, 75%, 50% and 25%. Marks are observations and lines are the quantile regressions. [Colour figure can be viewed at wileyonlinelibrary.com]

To validate the inferred effect of life cycle stage on observed size, we tested differences between stages for their respective slopes and intercepts estimated for further quantiles, namely the 25%, 50% and 75% quantiles (Fig. 7). The slopes estimated for these quantiles never differed among stages (*P* > 0.322 based on permutation test with 2,000 randomizations). Intercepts did not differ among stages for the 75% quantiles (*P_M_*_≠_ *_F_* = 0.275, *P_M_*_≠_ *_D_* = 0.116 and *P_F_*_≠_ *_D_* = 0.063). Intercepts differed among stages for the 50% quantiles with a higher intercept for females compared to the diploid intercept (*P_M_*_≠_ *_F_* = 0.084, *P_M_*_≠_ *_D_* = 0.427 and *P_F_*_≠_ *_D_* = 0.037). Intercepts estimated for the 25% quantile showed that the diploids’ intercept was lower than the ones of both haploid stages (*P_M_*_≠_ *_F_* = 0.0765, *P_M_*_≠_ *_D_* = 0.0315 and *P_F_*_≠_ *_D_* = 0.0095). Despite the punctual significant differences, the quantiles closer to the central tendency tended to differentiate less among stages (Fig. 7), strengthening the idea that all fronds tended to break to approximate sizes.

**Fig. 8:**
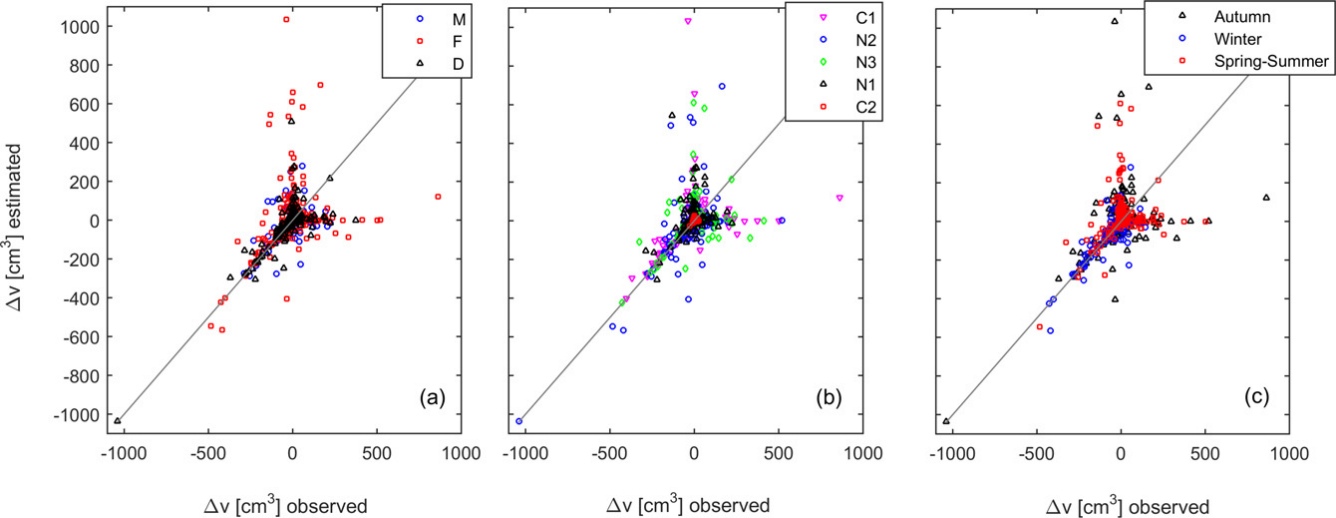
Model validation matching the predicted and observed change in frond volume. [Colour figure can be viewed at wileyonlinelibrary.com]

Validating this general model, the forecasted size increments on average matched the size increments actually observed (Fig. 8). Still, a clarification about the validation is required: while the growth rates were modeled by a deterministic algorithm, the breakage rates were modeled by a stochastic algorithm where the size of the broken fragments was randomly sampled from a *Β* distribution. Consequently, although the probability distributions of the effective and forecasted frond loss matched, on an individual basis this was impossible. Three situations occurred, then: (i) both effective and forecasted frond losses were minor or inexistent, with the net size increment resulting mainly from frond growth rates. In this case, the observed and forecasted size increments (∆*x*) tended to be similar. This corresponds to the data plotted in the filament over the 1:1 gray line, validating the model component relative to growth. However, the stochasticity in the breakage algorithm could lead either to (ii) the effective frond losses were much larger than the forecasted. In this case, the observed and forecasted size increments (∆*x*) were uneven, with the data being plotted in the filament departing the 1:1 line upwards, or (iii) the effective frond losses were much smaller than the forecasted. In this case, the observed and forecasted size increments (∆*x*) were uneven, with the data being plotted in the filament departing the 1:1 line horizontally to the right. The 1:1 diagonal is an axis of symmetry to the scattered data suggesting that the breakage algorithm forecasts a breakage probability distribution similar to the observed. Females extended longer along both x and y axes Fig. 8a); meaning that they were both observed and simulated growing more than the remaining stages. Fronds in C2 extend less along both axes (Fig. 8b); meaning that they were both observed and simulated growing less than the fronds in the remaining sites. Fronds during winter extend less along both axes (Fig. 8c); meaning that they were both observed and simulated growing less than during the remaining seasons.

## DISCUSSION

Our results show that *Agarophyton chilense* haploid males grow at least as much as diploids, while haploid females attain maximum sizes about twice as large as that of diploids, and that the maximum exponential growth rate (i.e., without size‐dependent limitation) of haploid females is roughly 15% larger than of diploids. In another experiment surveying *Sarcothalia lanceata* individuals yearly round in nutrient‐limited populations in New Zealand, Neill et al. (2018) observed that haploid males grew faster than diploids whereas haploid females attained larger sizes. However, contrasting with the present results, faster growth of apical meristems in diploid than in haploid fragments has been reported in a short‐term lab experiment also with *A. chilense* (Guillemin et al. 2013). This inconsistence with the growth dynamics determined from surveys of entire individuals may result from fronds not growing exclusively from their tips. Indeed, the meristem may extend beyond the apex and along the whole thallus (Hommersand et al. 1993). An alternative explanation is the lack of the holdfast. In kelp species the holdfast regulates growth to fit the environmental seasonality by activating and inactivating growth meristems (Luning 1993). It may be that, in the isomorphic biphasic life cycle of red algae, the haploids are more dependent on holdfast stimulus than the diploids. Alternatively, this could be due to the contrast between the stable environment of the laboratorial experiment by Guillemin et al. (2013) and the harsh and unstable environment during this 2‐y‐long survey. Coping with desiccation and UV requires costly protective metabolites (Molina and Montecino 1996, Jiang et al. 2008, Cruces et al. 2018), besides the negative impacts on photosynthetic efficiency (Ganzon‐Fortes 1997, Sagert et al. 1997, Silva et al. 1998, Gévaert et al. 2003, Gomez et al. 2004, Varela et al. 2006), resulting in an overall negative impact on growth (Andrés et al. 2006). Hence, the Corral and Niebla sites, as well as the different seasons, present different challenges to photosynthetic efficiency, resource use and potential for growth of red algae. The observed marked differences in *A. chilense* growth (this study) and survival (Vieira et al. 2018a) between the Corral 2 site and the remaining sites suggest that *A. chilense* demography is strongly conditioned by stress from desiccation and UV exposure, and the metabolic costs of coping with them. This has already been generalized for red algae from these locations (Molina and Montecino 1996, Gomez et al. 2004, Andrés et al. 2006, Varela et al. 2006, Cruces et al. 2018).

Intra‐specific competition in non‐clonal plants and seaweeds has classically been associated to the removal of weaker and smaller individuals by the stronger and larger ones, the latest typically growing and keeping the stands critically crowed (Weller 1987a, Weller 1987b, Scrosati 1997, 2005, Creed et al. 1998, 2019). Larger fronds not only inhibit growth of the smaller of the same cohort but also of germlings from new cohorts (Steen and Scrosati 2004). The *Agarophyton chilense* stands showed a different dynamic: as the stands got denser but the winter season arrived, the largest fronds, although surviving more than the smaller ones (Vieira et al. 2018a), did not grow (although the smaller fronds did) but shrunk instead (this study). This observation suggests that at least two environmental factors determinant for growth were acting jointly (see Atkinson and Smith 1983 for growth dependency on multiple factors). While one was promoting generalized growth the other was a limiting factor setting a maximum body size that could be sustainably maintained and beyond which larger fronds shrunk. Shrinkage of larger *A. chilense* fronds and holdfasts had already been observed in the laboratory, with bleaching and cell senescence in response to lower salinity and light availability (figs. 3 and 4 in Guillemin et al. 2013). Moreover, in this case the diploids were more prone to stress. Frond shrinkage was also observed in *Gelidium sesquipedale* along the Portuguese coast during winter (Santos 1994), in *Acanthophora spicifera* in Brasil (also reared in the laboratory) as a response to salinity stress (Pereira et al. 2017), and in yearly round nutrient‐limited *Sarcothalia lanceata* populations from New Zealand (Neill et al. 2018).

The breakage of *Agarophyton chilense* fronds was predominantly size dependent, matching previous algal studies (Santos 1994, Mach et al. 2007, 2011). This could be expected as breakage is mainly induced by drag (from waves and/or currents) on the fronds (Mach et al. 2011). Our data further suggested that, in *A. chilense* fronds, breakage is independent from life cycle stage, age, maturity state or season. The absence of a seasonal effect is not surprising since the sampling sites were located in a sheltered part of the coast, and thus under limited influence of waves and their seasonality. Nevertheless, failure by fatigue has been demonstrated important for frond breakage, and dependent on other seasonal stressors as temperature (Mach et al. 2007, 2011). Our complementary tests following the methodology by Cade and Noon (2003) show that lower quantiles for the size increment distribution tend not to differ, or to differ little, among stages. This confirmed that, irrespective of life cycle stage, similar‐sized fronds tend to break by similar amounts and thus to similar sizes. This finding supports the idea that females break more only when/because they attain larger sizes. A lab experiment testing for breakage of *A. chilense* fronds will be needed to verify the conclusions drawn from this field work. Previous reports from red algae are few and do not allow to easily draw general conclusions. The absence of ploidy differences in breakage rates was observed in *Chondrus crispus* (Carrington et al. 2001). In *Mazzaella flaccida* (Mach et al. 2011), the absence of ploidy effects is also evident in the shorter size classes. However, in the larger size class, diploids broke on average less often than haploids. Large variances around these means have been reported and the significance of their differences was not tested (Mach et al. 2011). A lab experiment on a species of the same genus as *A. chilense*, *A. vermiculophyllum* (i.e., reported as *Gracilaria vermiculophylla* at the time) showed gametophytes easier to debranch than tetrasporophytes (Lees et al. 2018). On the contrary, Bellgrove and Aoki (2020) report that tetrasporophytes of *Chondrus verrucosus* exhibit weaker tissue strength and attachment than gametophytes. Using the same *A. chilense* data set, we previously demonstrated that, besides growing better, haploid females also survive better than diploids (Vieira et al. 2018a). Better survival of haploids than diploids was also demonstrated in a previous laboratory experiment with *A. chilense* (Guillemin et al. 2013) as well as in other macro‐algal field experiments (Olson 1990, Thornber et al. 2006, Vergés et al. 2008). The haploids also include males, whose lesser growth (this work) and survival (Vieira et al. 2018a) mismatches female performance in *A. chilense*. However, males were almost always fecund, even during the spring‐summer when many females and diploids ceased fecundity (Vieira et al. 2018b). Since in *A. chilense* fecundity is proportional to frond size, the additional growth effort of males during the winter (this work) is fundamental for their high fecundity (Vieira et al. 2018b). This additional effort helps maintaining larger male fronds in the population and could support a non‐limiting supply of gametes to fertilize females (Engel et al. 1999). In fact, during spring‐summer, approximately 40% of the medium‐sized and large‐sized females in Niebla, and approximately 40% of the medium‐sized and 100% of large‐sized females in Corral, were fertile (fig. 2, Vieira et al. 2018b). Although this was a significant decrease relative to other seasons, when approximately 100% of the females were fertile, it still represented numerous fertile medium‐sized and large‐sized females. Given that fertility was proportional to frond size and that spring‐summer was when fronds attained larger sizes, the spring‐summer production between 70 and 1,000 carpospore per cm^3^ of female gametophyte thallus in the environmentally milder Niebla location resulted in an overall carpospore production not much different from the overall carpospores production at Niebla during the remaining seasons (fig. 3, Vieira et al. 2018b). The determinant influence of growth rates on reproductive output and ploidy field abundances had already been theoretically demonstrated by Vieira and Santos (2010, 2012a). From all these evidences, we propose that haploids have more available resources to spare on survival and growth (i.e., females) or reproduction and winter growth (i.e., males) than diploids, maybe because of the lower maintenance costs of their haploid genome.

The resource limitation hypothesis revolves around the added cost of larger amounts of genetic information, and not about the quality of the information encoded by different alleles. Applied to the evolutionary stability of biphasic life cycles, it is about the cost of haploidy vs. diploidy constraining the life cycle structure; and not about different alleles encoding morphologically or eco‐physiologically different individuals of the same ploidy stage. The selection and evolution of the biphasic life cycle starts with the emergence of “mutant” alleles encoding for one of the ploidy phases to be reduced or bypassed. The occurrence of the related “mutant” phenotypes has been observed (Tokida and Yamamoto 1965, Bird et al. 1977, Maggs 1988, Destombe et al. 1989). It is the relative fitness of “ordinary” vs. “mutant” alleles that must be compared (Richerd et al. 1993, Hughes and Otto 1999, Hall 2000). A resource limitation hypothesis must demonstrate that, relative to alternative reduced versions of the life cycle, enduring the full life cycle optimizes the cost of DNA production and maintenance, with the application of the spared resources benefiting the probability of passing to future generations “ordinary” alleles encoding for the full life cycle. Such benefits may come in a diversity of ways: (i) haploid females survive more when resources are scarce and competition is strong (Vieira et al. 2018a), (ii) haploid males are more fecund during the whole seasonal cycle (Vieira et al. 2018b), (iii) haploids grow larger and faster (this work), thus increasing fertility, since gamete and spore production is proportional to frond size (Vieira et al. 2018b), and (iv) the diploid germlings survive more (Vieira et al. 2018a), probably benefiting from the maternal care by the haploid females passing‐on to their diploid progeny the resources and metabolites enhancing survival (Kamiya and Kawai 2002). The effectiveness of this maternal care is crucial. No other diploid advantage was found in situ in *Agarophyton chilense* natural stands besides enhanced germlings’ survival, and the simultaneous existence of at least one diploid advantage is fundamental both for the advantage of the “ordinary” alleles and to ground conditional differentiation as the driver of the evolutionary stability of isomorphic biphasic life cycles.

The knowledge brought about by this study can be applied by *Agarophyton chilense* farmers. Farming is traditionally done by burying frond fragments into soft‐bottomed bays and growing them vegetatively (Guillemin et al. 2008). A couple of our findings are particularly relevant in the farming context. The best season to plant a new stand depends on the initial size of the fragments and how much they can grow until winter, a season during which the larger fronds become severely constrained. Considering only biomass yield, our results point out that the most efficient strategy is to pick only large fronds, thereby freeing up space to let the smaller fronds grow. However, the threshold size for picking fronds cannot be much larger than 200 cm^3^, as in this case only the haploid females would be systematically harvested (see Fig. 2); soon leaving the stand dominated by males and diploids that can hardly grow beyond such size and therefore rarely be harvested. Picking up large fronds should take place year‐round as fronds grow fast but break often, besides having short life spans. Above all, large fronds should be picked before the start of the winter season as, even if they do not break or die, they will surely shrink. Thus, waste of biomass is inevitable if these fronds are not harvested ahead of winter.

## MATERIALSANDMETHODS

This research was supported by CONICYT (Fondo Nacional de Desarrollo Científico y Tecnológico FONDECYT) under grant number 1090360 and 1170541. VV was supported by FCT/MCTES (PIDDAC) through project LARSyS ‐ FCT Pluriannual funding 2020‐2023 (UIDB/EEA/50009/2020). AHE was supported by fellowships SFRH/BPD/63703/2009 and SFRH/BPD/107878/2015 and through project UID/Multi/04326/2020 of the National Science Foundation FCT of Portugal. OH was funded by National PhD Degree Scholarships, CONICYT. The funders took no part in the design of the study, in the collection, analysis, and interpretation of data, and in writing the manuscript. Our gratitude to the anonymous reviewers for their effort and contribution to improve this article.

## AUTHOR CONTRIBUTIONS

**V.M.N.C.S. Vieira**: Conceptualization (lead); data curation (equal); formal analysis (lead); funding acquisition (equal); investigation (lead); methodology (lead); software (lead); supervision (lead); validation (lead); visualization (lead); writing‐original draft (lead); writing‐review & editing (equal). **A.H. Engelen**: Conceptualization (supporting); formal analysis (supporting); funding acquisition (supporting); investigation (supporting); visualization (supporting); writing‐review & editing (equal). **M.L. Guillemin**: Conceptualization (equal); data curation (lead); formal analysis (supporting); funding acquisition (lead); investigation (supporting); methodology (lead); project administration (lead); resources (equal); supervision (equal); validation (supporting); visualization (supporting); writing‐review & editing (equal). **O.R. Huanel**: Data curation (lead); funding acquisition (supporting); investigation (supporting); methodology (supporting); project administration (supporting); resources (supporting).

## Supporting information


**Figure S1**. Corral and Niebla sampling sites. (A) Location of the Corral and Niebla sampling sites within the Valdivia River Estuary. (B) and (C) Niebla permanently submerged rock‐pools during low tide. (D) Corral site located on rocky platform presenting a gentle slope, here at the beginning of the low tide. All photos O. Huanel and M. L. Guillemin.
