# Supplementary figures and images for: Differential Frond Growth in the Isomorphic Haploid–diploid Red Seaweed Agarophyton chilense by Long‐term In Situ Monitoring
Source: J Phycol. 2021 Feb 9;57(2):592–605. doi: 10.1111/jpy.13110 (PMC8247958; doi:10.1111/jpy.13110)

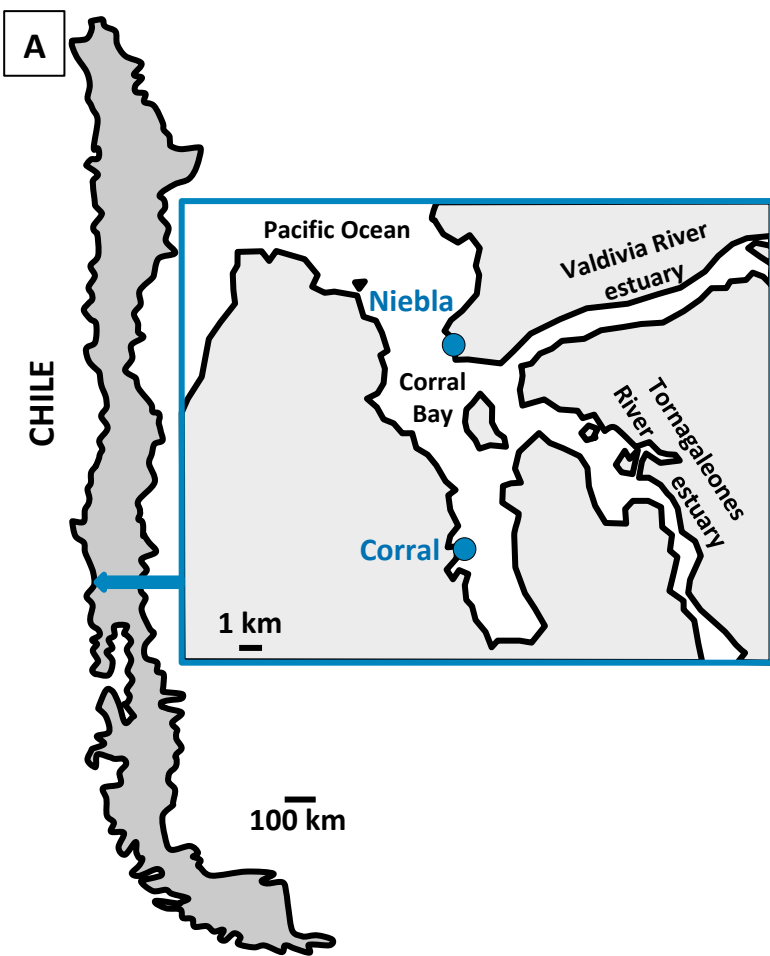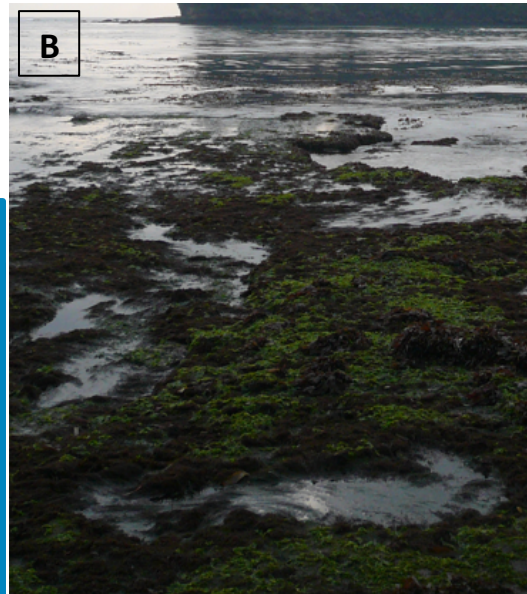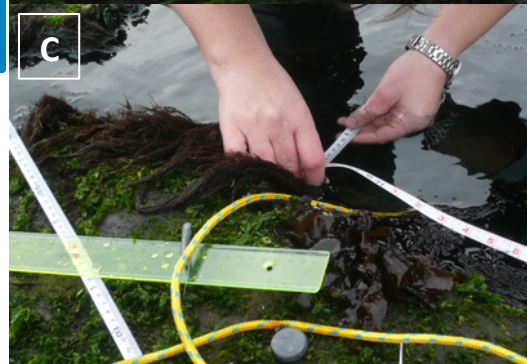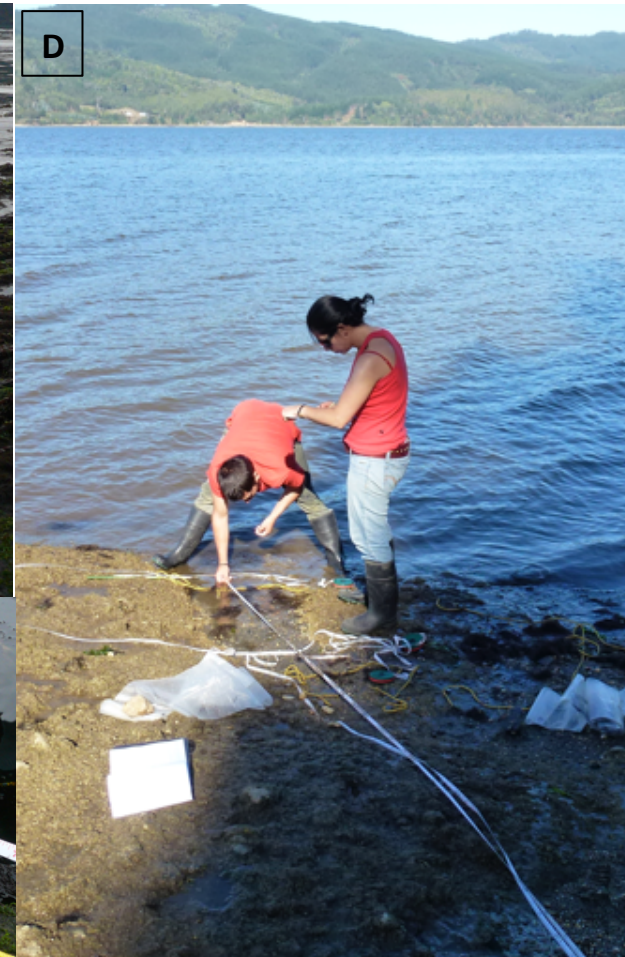

Supplement: Supplementary file 1 — Figure S1. Corral and Niebla sampling sites. (A) Location of the Corral and Niebla sampling sites within the Valdivia River Estuary. (B) and (C) Niebla permanently submerged rock‐pools during low tide. (D) Corral site located on rocky platform presenting a gentle slope, here at the beginning of the low tide. All photos O. Huanel and M. L. Guillemin. [file JPY-57-592-s001.pdf]
